# On the Use of Colchicum in Erysipelas

**Published:** 1834-04-01

**Authors:** John Bullock


*To the Editor of the Medical Quarterly Review.*

Sir: Not being aware that colchicum has ever been used as the principal remedial agent in the treatment of erysipelas, I am induced, through the medium of your excellent work, to communicate to the profession the result of some cases so treated, that have come under my actual observation. Its mode of action is the question; its efficacy is indubitable. The relief derived from colchicum in rheumatic and gouty affections is generally accompanied with nausea, sometimes vomiting, griping, and a copious and frequent discharge from the bowels. Such, however, is not invariably the case, as many of the most rapidly decisive cases of the two latter affections terminating successfully that I have witnessed have been wholly unattended by any symptom of the kind. How then is its action in erysipelas to be accounted for? In my opinion, principally through the medium of the arterial system; and for the following reason.

I have upon all occasions observed the subsidence of erysipelatous inflammation accompanied by an equal diminution in the force and frequency of the pulse, which becomes soft and regular, without any jerking or intermission whatever. Such being the case, I can readily conceive why, the heart's action being subdued, a proportionably small quantity of blood is propelled into the capillary arteries, and a relative diminution in the cuticular inflammation is the result.

Bleeding has also been recommended and practised by many of the most eminent men in our profession in the acute inflammatory stage, and acts in the same manner as colchicum by lessening the vascular excitement, while the latter possesses a peculiar influence in controlling the most violent disturbance of the arterial system, without at the same time encroaching on the restorative powers of the system; the former in accomplishing this purpose produces a tendency to a train of low typhoid symptoms, from which it is often impossible to rescue the patient, even with the aid of external heat and the exhibition of internal diffusible stimuli; consequently, it becomes a great desideratum to know by what means we can safely combat the intense burning heat, redness, pain, tumefaction and inflammation supervening on an attack of erysipelas without incurring the risk of running into the consecutive typhoid stage; and this object can be safely and surely obtained, I have no hesitation in saying, by administering colchicum in properly regulated doses. In confirmation of these views, I will now lay before you the cases before alluded to.

R. Berry, ætat. twenty-eight, admitted November 21, 1833, with rheumatism. On the 22d his scrotum became very highly inflamed, œdematous, and enlarged to the size of a child's head. He had been previously well purged. Several veins in the scrotum were opened with a lancet, and bled freely into a warm linseed-meal poultice.

23d. Swelling of scrotum very much increased, erysipelatous inflammation extending down both thighs up over the abdomen and left side of the face and neck; skin of a scarlet or crimson hue; smart burning heat; pulse 140; tongue dry and very white; bowels open; great thirst.

Four P.M. ℞. Pulv. Colchici, gr. xv.; Sodæ Carbon. ℥i. M. ut ft. pulv. statim. sumendus.

In about a quarter of an hour, the frequency of pulse, redness, and burning heat of skin very sensibly diminished, and the patient at the same time said that he was cooler and much more comfortable. At the expiration of an hour, the heat and crimson hue of the skin, the frequency and fulness of pulse augmented considerably.

Five P. M. Pulv. Colchici gr. x.; Sodæ Carbon. ℈ij.; M. ut ft. pulv. statim. sumendus.

This dose was followed by effects as beneficial, but more lasting, the efflorescence and other symptoms not recurring so rapidly or in so aggravated a form. It was necessary to repeat the last powder at seven o'clock. This had the desired effect of thoroughly subduing the frequency and fulness of the pulse, the heat and redness of the skin, and swelling of the scrotum.

These large and frequently repeated doses of colchicum so rapidly and decidedly reduced the action of the pulse, that it became an imperative duty to watch its effects with the most scrutinizing attention, and delay the exhibition of another dose till a tolerably well established reaction took place. To watch the ebb and flow of symptoms between the intervals of giving the colchicum was both highly interesting and satisfactory; for, in the short space of five or six hours, the patient was restored to a perfect state of health.

25th. The swelling, together with the redness of the scrotum and face, had nearly subsided, the skin was cool, tongue clean, pulse eighty, soft. ℞. Acid Hydrocyanici dil. m. ij.; Vin. Colchici, m. xv.; Aquæ ℥i.; M. ut ft. haustus, tertiis horis sumendus.

On the morning of the 26th not a vestige of erysipelas remained.

II. Charles Mace, æt. nineteen, admitted November 29th, with great determination of blood to the head, and generally plethoric habit, was much relieved by active purgative medicines. December 9th, complains of sore throat; face much flushed.

Dec. 10th. Much pain, great tumefaction, and high erysipelatous blush over the nose and round the orbits; had but very little sleep the night previous, owing to the burning heat of his face. Pulse 108, tongue furred, bowels open from previous medicine.—The face was covered with flour. VS. ℥xij. Vin. Sem. Colchici, ʒij.; Sodæ Carb. ʒss.; P. Rhæi, ʒss.; Mist. Camph. ℥viij. ℥j. ter die.

11th. Erysipelas spreading; pulse ninety-four, hard. The blood was cupped.—Rep. mist. quartis horis.

12th. The tumefaction was so much increased, that the palpebræ are nearly closed; heat of face much increased; pulse ninety-six.—Omitt. mist. Sodæ Carb. ℈i.; P. Colch. gr. v. quartis horis.

13th. Erysipelas is receding from the nose and eyes, but spreading over the cheeks; pulse ninety, soft.—Rept. pulv. secundis horis. Hirudines xij.

14th. Efflorescence is disappearing fast; pulse ninety-two, soft.—Sodæ Carb. et Acid. Tart. in statu effervesc. p. r. n.

16th. The erysipelas is disappearing very fast; pulse ninety, soft.—Omitt. Pulv. Colchici.

17th. Quite free from erysipelas; pulse ninety-six, soft; skin dry.—Baln. tepid. Port wine ℥iv., mutton chop daily.

19. Continue chop and wine. Dec. Cinch. ℥j. ter die.

24. Omitt. Vin. Lusitan. Two pints of porter daily. Quin. Sulph., Ferri Sulph. *aa* gr. xvj.; Acid. Sulph. d. ʒj.; Mist. Camph. ℥viij. ℥j. ter die. He only complains of weakness.

30th. Dismissed, perfectly well.

III. October 23d. David Simmons, æt. thirty-seven, an emaciated and feeble subject, was admitted for ascites.

December 21st. An erysipelatous inflammation appeared over the whole abdomen, in addition to his dropsy, which remained unrelieved. Pulse 124.—P. Colch. gr. v.; Sodæ Carb. gr. xv. quartis horis.

22d. Sore throat better; efflorescence extending down the inside of both thighs; pulse 136, small; tongue furred; much thirst.

24th. Erysipelas has entirely left the abdomen. Omitt. Pulv. Colchici. Pulse 120, soft; bowels freely open.—Vin. Ant. Tart. ℥ss.; Liq. Ammon. Acet. ʒij.; Mist. Camph. ℥ij. ℥j. quartis horis.

On the 27th, the erysipelas returned with great redness, tumefaction, heat, and pain over the whole face, particularly round the eyes and nose; bowels confined.—Calomel. gr. v.; Ext. Coloc. gr. x. statim. Haust. Cath. post duas horas.—Pulse 136, feeble.

28th. Erysipelas has spread over the whole face, the palpebræ being particularly swollen and painful; great heat of skin.—Baln. tepid. Vin. Colch. ʒij.; Vin. Ant. Tart. ℥ss.; Mist. Camph. ℥vijss. ℥j. quartis horis.

29th. The erysipelatous blush is fading fast; pulse 128, small; tongue very red, but clean; bowels regular.

31st. Omitt. Mist. Colch. He was ordered to take Ammon. Carb. in excess, with Acid. Tart. in effervescence, secundis horis.

January 1st. Free from erysipelas; pulse 130; tongue clean, and red; bowels open.

It is worthy of remark, that the dropsy obstinately resisted, both before and after the attack of erysipelas, the most powerful diuretics and hydragogues that could be exhibited, viz. squills, digitalis, dec. pyrolæ umbellatæ, elaterium, potas. supertart., and many others. He is now fast recovering from the application of a large blister covering the whole abdominal region. He has now his third blister on, and is amazingly improved; in fact, quite well.

IV. John Sergeant, æt. thirty, a poor, emaciated, phthisical patient, was admitted, December 8th, with a cough of six months' standing, and thick, greenish, muco-purulent expectoration. After being in the hospital a few days, a very large cavity was discovered in the right subclavian region.

January 7th. After complaining of sore-throat, the upper part of his face became the seat of violent erysipelatous inflammation; pulse 110, hard; bowels open; tongue very dry.

—Haust. Effervescens. p. r. n. P. Colchici, gr. v.; Sodæ Carbon. gr. xv. quartis horis.

8th. Erysipelas extending over the whole face and scalp; palpebræ particularly swollen, and immoveably closed.—Pt. pulv.

9th. Pulse 128, soft and full. Erysipelas is fading. The bowels being much purged, he was ordered to omit the colchicum, and take an excess of Ammon. with Tart. Acid. and Tr. Opii, m. x. in a state of effervescence, secundis horis.

11th. Much better; appetite improving; pulse 102.

13th. Only complains of the eyelids being stiff; in the right a slight sloughing took place.

He left on the 24th, under the fallacious hope of getting well, but died from phthisis only a few days after.

v. Robert Sutton, æt. fifty, admitted December 10th, 1833, with an eruption extending over the cheeks and ears.

January 1st. Erysipelas on the left cheek and ear. On the 2d and 3d, it spread over the whole of the head, face, neck, and upper part of the thorax, the eyelids being greatly distended, exquisitely tender, and inflamed; skin very hot and dry; tongue furred; great thirst; pulse varying from 104 to 114, hard and full.—Calomel. gr. iij.; Pulv. Jalap. gr. xij. statim. Vin. Colchici, m. xv.; Vin. Ant. Tart. ʒss.; Aquæ, ℥j. secundis horis.

This medicine was continued till the 7th, with warm bath daily, and occasionally giving a small quantity of brandy; the frequency of the pulse, as well as the state of the erysipelas, fluctuating from time to time.

7th. Erysipelas has subsided; pulse 110, weak; skin cool; slightly delirious.—℞. Liq. Ammon. Acet. ℥iij.; Inf. Sennæ, ℥v. sumat ℥j. quartis horis. Cont. baln. tepid.

He obstinately refused to take either his medicine or food with any regularity, and was attacked with very slight patches of erratic erysipelas, till the 15th. Pulse feeble; tongue dry; bowels open; slight pain in the head at times.—Quin. Sulph. gr. ij.; Inf. Rosæ, ℥j. quartis horis. Wine and porter daily.

23d. Four P.M. The erysipelas has returned with increased violence, the whole of the head and face being enormously swollen, red, and œdematous.—Pulv. Colch. gr. x.; Sodæ Carb. ℈ij. statim.

Six P.M. Very much relieved; swelling subsiding rapidly. —Vin. Colch. m. xv.; Mist. Camph. ℥j. quartis horis.

Eleven P.M. Cont. Vin. Colch. sextis horis.

24th. Eight A.M. Pulse feeble. The erysipelas has entirely disappeared, and the patient is sinking. He refused to take brandy, or any other stimulus, and expired at twelve o'clock.

*Post-mortem examination, forty-eight hours after death.* The cellular membrane occupying the whole surface of the occipito-frontalis muscle was infiltrated with serum; a great quantity of which was also found between the membranes of the brain, which was slightly congested. The liver was of a nutmeg colour, and easily broken between the thumb and finger.

This is the only unsuccessful case I have yet seen treated with colchicum out of a great number, besides those recorded above.

I remain, sir, yours respectfully,

JOHN BULLOCK,

*Westminster Hospital.* Apothecary.

